# Can brain stimulation boost memory performance?

**DOI:** 10.1371/journal.pbio.3001404

**Published:** 2021-09-29

**Authors:** Yevgenia Rosenblum, Martin Dresler

**Affiliations:** 1 Donders Institute for Brain, Cognition and Behaviour, Radboud University Medical Center, Nijmegen, the Netherlands; 2 Sackler School of Medicine, Department of Neurology and Neurosurgery, Tel Aviv University, Tel Aviv, Israel

## Abstract

Memory performance plays a crucial role across the human life span, from early education to age-related decline. This Primer explores the implications of a new study in PLOS Biology which shows that verbal learning can be enhanced by applying repetitive transcranial magnetic stimulation over the left prefrontal cortex.

## Teaser

Memory performance is crucial across the human life, from early education to age-related decline. A new study in *PLOS Biology* found that verbal learning can be enhanced by applying repetitive transcranial magnetic stimulation (rTMS) over the left prefrontal cortex.

Remembering previous experiences and learning new information determine much of our mental life, from early childhood and formal education to age-related cognitive decline. Accordingly, humans have always striven to enhance their memory performance, from ancient mnemonic strategies [1] to modern neuroenhancement technology [2]. For example, deep brain stimulation of the hippocampus and surrounding structures of the medial temporal lobe has been shown to improve memory performance in neurosurgical patients [3]. Despite such promising findings, the invasive approach obviously has less translational appeal compared to noninvasive techniques, such as transcranial magnetic stimulation (TMS).

TMS produces fully reversible changes in brain activity by delivering magnetic pulses that pass through the skull to the area of interest [4]. The body of TMS research used to modulate memory constantly emerges and confirms that the dorsolateral prefrontal cortex is one of the key regions for working and episodic memory processing [4,5]. This area is thought to control sensory inputs represented and processed by the posterior cortex and mediate executive control, being involved in both the encoding and retrieval of episodic memories [5]. Several studies report that stimulation over the left prefrontal cortex improves memory performance in patients with depression and schizophrenia. In healthy individuals, however, such interventions have produced mixed and inconclusive results [5].

The new work by van der Plas and colleagues in this issue of *PLOS Biology* [6] takes an important step toward a deeper understanding of the effect of repetitive transcranial magnetic stimulation (rTMS) on memory encoding. Based on previous reports that fast (i.e., more than 5 pulses per second) prefrontal stimulation reduces memory, the authors assumed that prefrontal activity in the left dorsolateral prefrontal cortex has an inverse relationship to memory performance. In the current study, the authors applied slow rTMS over the left dorsolateral prefrontal cortex, hypothesizing that this procedure would suppress its activity and, thus, boost the memory performance.

**Fig 1:**
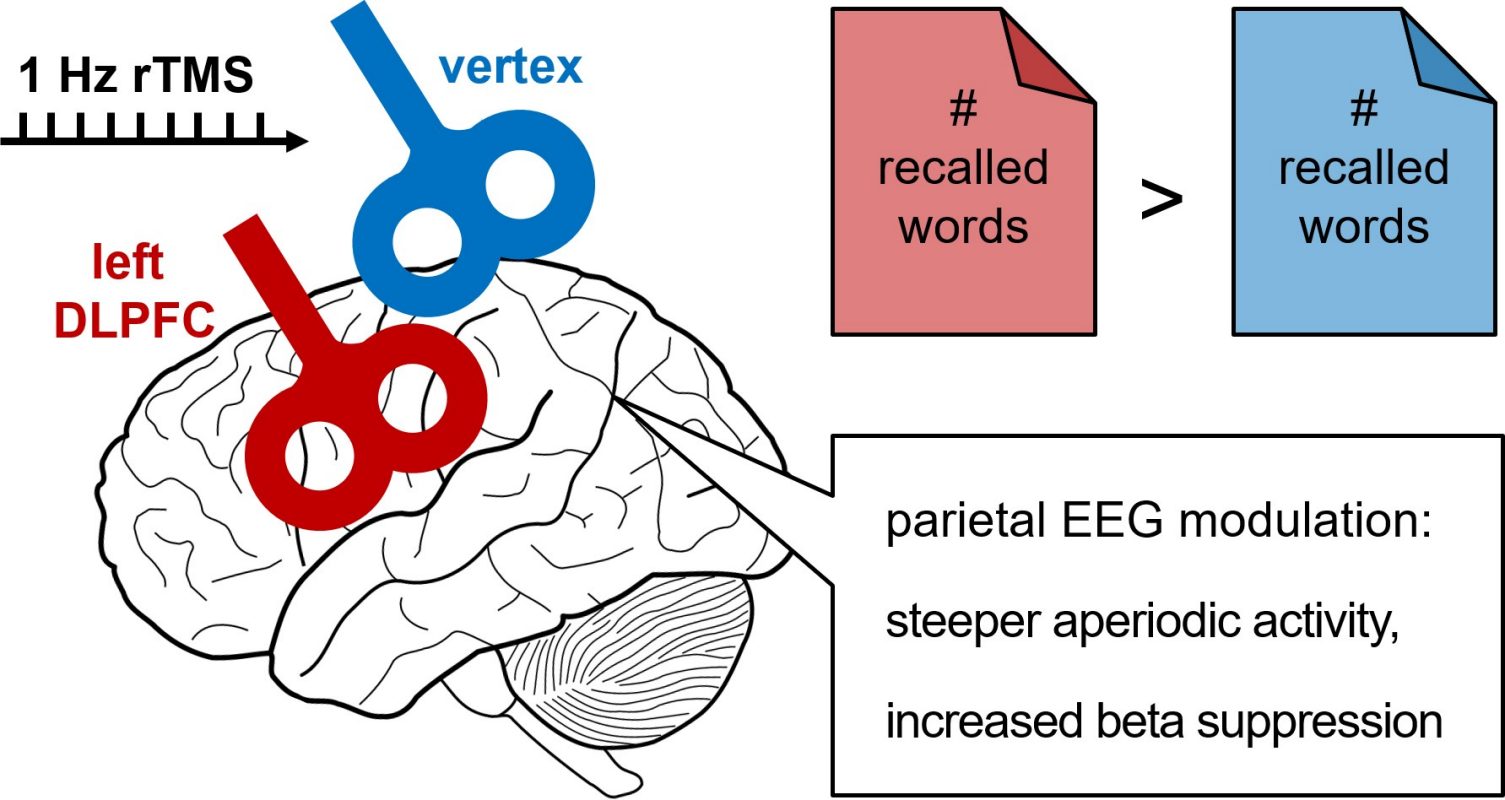
The effect of slow brain stimulation on different brain areas and memory performance. Applying slow (1 pulse per second) rTMS over the left DLPFC (compared to the vertex at top of the head as control region) is associated with enhanced word memory performance (10% to 20% increase). Apart from the effect of rTMS on memory performance, prefrontal stimulation causes enhanced event-related beta suppression at the parietal cortex, a neural response known to be a correlate of memory encoding. However, a further follow-up investigation reveals that increased beta suppression is driven by a steeper slope of aperiodic (scale free) rather than periodic (oscillatory) activity, a feature that reflects a decreased excitation to inhibition ratio of neural electrical currents. DLPFC, dorsolateral prefrontal cortex; EEG, electroencephalogram; rTMS, repetitive transcranial magnetic stimulation.

Specifically, each participant was presented with 2 lists of words and asked to keep these words in mind. When the participants were presented with the first list, no stimulation was applied. When the participants were presented with the second list, they received rTMS either over the prefrontal cortex or over the vertex (the top of the head, Fig 1), a control site thought to have relatively little influence over ongoing brain processes [7]. Shortly after this task, the participants were asked to recall all words just presented: Those who received prefrontal intervention recalled more words than those who received vertex stimulation. To ensure that this was not merely a chance finding, the authors replicated their results using a within-subjects design, where each participant received both prefrontal and vertex stimulation. Here, again, the participants recalled more words from lists in which they received prefrontal stimulation regardless of the word’s position in the list.

One of the most remarkable aspects of this study is the magnitude of the effects of prefrontal stimulation on memory: The increase in recall performance reached 10% to 20%, exceeding effects seen in most other TMS or even invasive electrical stimulation approaches with similar behavioral paradigms [3,4]. Of note, also the remarkable memory-boosting effects of mnemonic encoding strategies such as the “method of loci” have been related to decreased prefrontal activation [1].

Apart from the effect of rTMS on memory performance, the authors also explored how rTMS influences the neural activity of unstimulated areas. Interestingly, for the words that were later remembered, prefrontal rTMS enhanced event-related beta suppression at posterior areas. While the parietal cortex is hypothesized to have a role in attending to memory representations [4], beta suppression is a known correlate of memory encoding. A further in-depth analysis conducted in the current study revealed that enhanced beta suppression is driven by steeper aperiodic (scale free) rather than periodic (oscillatory) activity. Given that the slope of the aperiodic signal reflects the balance between excitatory and inhibitory electrical currents in the brain [8], the increased steepness of the aperiodic slope caused by prefrontal stimulation can be interpreted as an induced shift toward the predominance of neural inhibition. In other words, the prefrontal stimulation possibly propagates to the parietal cortex, applying an inhibitory effect to it. An alternative interpretation, proposed by the authors, suggests that prefrontal stimulation leads to enhanced functional connectivity between frontal and posterior regions and, as a consequence, enhanced stimulus processing and improved memory performance.

While the study results generally are in line with the assumption that slow rTMS has an inhibitory effect on neural activity, clearly directed inhibitory versus facilitative effects of slow versus fast rTMS are far from being a settled issue in the field. Given this disagreement, it would be interesting to test whether fast stimulation (considered to increase neural excitability) is associated with a flatter aperiodic slope, which, in turn, reflects a higher excitation to inhibition ratio.

To date, the importance of optimal balance between neural excitation and inhibition for flexible behavior, information processing, and memory encoding becomes increasingly recognized [9]. In this context, a deeper understanding of the effect of rTMS on the excitation to inhibition ratio can have important clinical implications, especially in the conditions linked to perturbations in this balance, such as epilepsy, autism, depression, and schizophrenia.

Considering the importance of memory function across the human life span, the current findings might have implications in educational or professional training settings where large amounts of information need to be memorized. Given that purely cognitive strategies for memory enhancement are often seen as more appropriate for young and healthy participants than direct stimulation of the brain [1,2], the study results might be of higher relevance for clinical contexts: Enhancing memory function might help patients with mild cognitive impairment, a population at high risk for developing Alzheimer disease and other types of dementia. So far, available studies that used rTMS in mild cognitive impairment yielded inconclusive results, ranging from “no effect” to “improved everyday memory for at least 1 month” [10]. This is likely due to several factors, most notably differences among the study designs and methods. The memory enhancement approach reported in the current study might open new strategies to counteract or delay the detrimental memory effects of dementia. No need to say that in the face of an aging society and the lack of treatments for age-associated neurological conditions, such options are highly needed.

However, both for healthy participants and dementia patients, one should keep in mind that rTMS can cause several side effects, including headaches, mood changes, tinnitus, and, in very rare cases, even epileptic seizures. Further, given that rTMS of a single site can influence entire brain networks, a better understanding of the mechanism of rTMS action is critical for identifying more precise targets for interventions in patients with psychiatric and neurological disorders. Another key question is whether the empirical results reported in the current study can be generalized to other memory processes—future research should assess whether the protocol reported here has an impact on memory tasks other than simple word learning. Despite these limitations, the memory enhancement approach reported in the current study appears to have promising potential, ranging from basic research to clinical interventions.
